# Comparison of enjoyment and energy expenditure of exergame with and without blood flow restriction in men and women

**DOI:** 10.1038/s41598-024-59379-8

**Published:** 2024-04-19

**Authors:** Zeynabalsadat Mousavi, Zohreh Karimi, Alexei Wong, Neda Cheraghloo, Hessan Bagheri, Reza Bagheri

**Affiliations:** 1 Nutrition and Food Service, Imam Khomeini Hospital Complex, Tehran University of Medical Sciences, Tehran, Iran; 2 Department of Physical Education and Sport Sciences, Science and Research Branch, Islamic Azad University, Tehran, Iran; 3 Department of Health and Human Performance, Marymount University, Arlington, USA; 4 Department of Epidemiology and Biostatistics, School of Public Health, Tehran University of Medical Sciences, Tehran, Iran; 5 Department of General Psychology, Islamic Azad University, South Tehran Branch, Tehran, Iran; 6 Department of Exercise Physiology, University of Isfahan, Isfahan, Iran

**Keywords:** Sedentary lifestyle, Sex characteristics, Energy expenditure, Physiology, Psychology, Health care

## Abstract

This study compared the effects of blood flow restriction (BFR) on intensity and perceived enjoyment during an exergame. Fourteen healthy young participants engaged in a boxing exergame for 20 min, with or without BFR, across two sessions. Perceived enjoyment levels were assessed using the Physical Activity Enjoyment Scale. Heart rate was monitored, and energy expenditure (EE) during exercise was calculated. A mixed model analysis of variance with repeated measures was used to evaluate differences in EE and enjoyment between exergame conditions (with and without BFR) as well as the interaction effects of these protocols with gender. Although not statistically significant, perceived enjoyment decreased with BFR inclusion for both genders. No significant differences were observed between men and women for both protocols. Regarding EE, there was no significant difference between the two groups (with and without BFR). However, a significant main effect of gender was found, with men exhibiting higher EE values in both protocols compared to women. In conclusion, exergames incorporating BFR impact perceptual responses, particularly perceived enjoyment. Furthermore, significant gender differences in EE were found, with men displaying higher values.

## Introduction

Sedentary behavior is a widely recognized risk factor for chronic diseases^1,2^, and its impact has been exacerbated by the increasing reliance on technology for leisure activities, including video games^3–5^. Indeed, several studies have demonstrated a consistent relationship between self-reported sedentary and screen time and increased all-cause mortality^6–9^. While sedentary activities such as video gaming have traditionally been associated with inactivity, the integration of physical activity into video games, known as active video games or exergaming, has the potential to promote physical activity and help meet recommended activity levels^10–14^. This innovative approach has shown promise in promoting physical activity across various populations^15^.

Exergames are entertaining video games designed to make exercise more enjoyable, requiring players to engage in light to moderate-intensity physical activity^11–14,16^. For instance, the Xbox with Kinect system enables players to freely move their bodies without the need for additional accessories, making it the first gaming system to allow unrestricted body movement^4^. Enjoyment (defined as a positive emotional state characterized by feelings of pleasure, liking, and fun) of physical activity is a crucial determinant of activity levels, particularly in young individuals^14,17,18^. Although this technology is relatively new, it has shown promising outcomes, including high participation rates, increased enjoyment, motivation, and fun compared to other forms of exercise^4,11,13,14^. Psychological factors, such as enjoyment and motivation, may play a pivotal role in the physiological outcomes associated with exergaming^11,13,14,19^. In multiplayer exergames, individuals are motivated to play more, as team-based gameplay generates excitement, promotes social interaction, and leads to greater energy expenditure^20,21^. Social interaction can enhance enjoyment, perceived pressure, and self-efficacy in group activities, with a positive correlation between social interaction and participation in exergames or physical activities^11,13,14,22^. In fact, measurements of physical activity intensity, such as the rating of perceived exertion (RPE) and energy expenditure (EE), have demonstrated that exergames elicit physiological intensity levels comparable to moderate and vigorous physical activity in healthy adults^10^. Notably, however, previous research has also indicated that the enjoyable nature of exergaming can lead to a lower RPE during physical activity performed at a moderate-to-vigorous intensity^15^.

Blood flow restriction (BFR) exercise is a distinctive approach that involves using low-intensity loads for resistance and aerobic exercises^23^. It has been found to elicit comparable improvements in certain physiological parameters to those achieved with moderate and high loads^24^. Consequently, BFR exercises have gained significant attention within the exercise and sports science community^23,25^. However, previous studies have indicated that BFR exercises result in heightened responses to perceptual parameters^26–34^. Specifically, perceptual responses such as increased perceived exertion (e.g., RPE) and leg discomfort were found to be more pronounced during low-intensity resistance exercise with BFR compared to without BFR^27–29,34^. Additionally, Silva et al. determined that mood states decreased following low-intensity resistance exercise with BFR, whereas this effect was not observed after low-intensity resistance exercise without BFR^35^. Similarly, studies conducted by Silva et al. also demonstrated that perceptual responses elicited by low-intensity aerobic exercise with BFR were more pronounced than those induced by low-intensity exercise without BFR and were similar to the responses observed during high-intensity aerobic exercise^32,33^. Moreover, previous research has reported that the extent of changes in perceptual parameters, including those related to exercise adherence, induced by exercise may be partially dependent on the magnitude of certain physiological responses, such as cardiovascular (e.g., heart rate [HR]) and metabolic (e.g., blood lactate) responses, during the exercise^36,37^. These previous findings suggest that aerobic and resistance exercises with BFR negatively impact perceptual responses, potentially decreasing adherence to both exercise modes among certain individuals^38^. Despite this knowledge, no study to date has investigated the detrimental effects of BFR exercises on major perceptual parameters (e.g., effect, task motivation, and enjoyment) that are associated with exercise adherence. Moreover, previous investigations have not explored the physiological responses to exergames incorporating BFR, nor have they examined potential sex differences in these responses. Understanding these responses is crucial, as previous studies have suggested that sex may influence the responses to exergames (without BFR)^15,39,40^. Consequently, this study aimed to compare the responses in intensity and perceived enjoyment resulting from a session of boxing exergame under two conditions: with and without BFR, and among both men and women. The objective was to investigate whether BFR can enhance the training intensity and determine its effect on the perceived enjoyment of the exergame, as well as to explore potential differences in these responses between men and women.

## Materials and methods

### Participants

Fourteen young and healthy participants (female [n = 8], male [n = 6]; age = 29.9 ± 7.04 years; BMI = 22.4 ± 3.46 kg m^−2^), who did not use any medications, participated in two separate sessions in this study (Table 1). The evaluation of participants’ health status encompassed a thorough assessment of their general physiological state and wellness. This assessment included an analysis of their medical history and an examination for the presence of diseases. Additionally, their consumption of alcohol and drugs, dietary habits, and levels of daily physical activity were meticulously scrutinized. The physical Activity Readiness Questionnaire (PAR-Q) and medical health questionnaire were used to collect the required data^41^. The participants were considered non-athletes (no recent participation in organized sport), had not partaken in a regular exercise program within the past year, and were not limited in their ability to participate in the boxing exergame exercise protocol. Participants were instructed to maintain regular sleep patterns and activities of daily living, avoid strenuous physical activity, dietary supplements, medication, cocoa, coffee, caffeinated beverages, alcohol, and tobacco for up to 48 h prior to boxing exergame sessions and data collection. In addition, while participants were instructed to maintain their usual dietary habits, they were also asked to consume a light dinner the night before the exercise test^42^. This was verified by reviewing their 24-h dietary recall questionnaires. It was confirmed that the caloric intake of all participants on the day prior to the test matched their required intake for weight maintenance, ranging from 1600 to 2200 kcal. They were also provided a breakfast of similar caloric value at the testing site, in a controlled environment. This breakfast included two slices of toast, a slice of sausage, and Gouda cheese, amounting to approximately 32 g of carbohydrates, 10 g of protein, 12 g of fat, and a total of 300 kcal. Moreover, participants reported no official clinical diagnoses pertaining to depression, anxiety levels, or other mental health disorders. To evaluate anxiety, the State-Trait Anxiety Inventory (STAI) was administered^43^. Following comprehensive explanations of the research procedures, participants provided informed consent in the presence of a witness to participate in the study. The protocol was reviewed by the Institutional Human Subject Committee and the Ethics Committee of the Islamic Azad University Science and Research Branch, Tehran, Iran (IR.IAU.SRB.REC.1399.100) and carried out in accordance with the Declaration of Helsinki^44^.

**Table 1:** Characteristics of participants.

	Age (year)	Height (cm)	Weight (kg)	BMI (kg m^−2^)
Sex				
Women	7.0 ± 28.8	5.0 ± 165.2	7.2 ± 57.8	2.1 ± 21.1
Men	7.3 ± 31.3	3.6 ± 179.5	13.1 ± 77.5	4.3 ± 24.1

Data are mean ± SD.

### Study design and exercise protocol

This investigation utilized a crossover design, encompassing 14 participants who were randomly assigned to two groups: one subjected to a boxing exergame with BFR, and the other to an identical exergame without BFR. The study commenced with a session of both groups partaking in the boxing exergame. This was followed by a 1-week washout phase, after which the groups switched conditions for the second week, thereby replicating the exercise regimen. Preceding the study’s initiation, participants were bifurcated into gender-specific cohorts, within which they were further randomized and paired. Participants were instructed to visit the designated site 1 week prior to the commencement of the initial protocol in order to get familiarized with the environment, devices utilized, and training regimen. Baseline anthropometric measurements, including height and body mass, were ascertained a week prior to the protocol commencement, utilizing a Seca 206 wall-mounted stadiometer (SECA, Germany) and a BF800 Beurer digital weight scale (Beurer, German), respectively.

Participants were instructed to report to the laboratory at 7 am to initiate the completion of the protocols. All assessments were conducted in the morning (8:00–11:00 am) under controlled ambient laboratory conditions (temperature: 24 °C; humidity: 42%). The study consisted of two training protocol sessions separated by a 1-week interval. At the start of each session, participants’ body temperature was measured to ensure the absence of fever or internal infection. Resting blood pressure (BP) was evaluated in the supine position in the morning prior to exercise testing, using Microlife BP A100, a digital sphygmomanometer (Microlife, Germany), with measurements taken thrice and averaged. Relative arm occlusion pressure was defined as being between 15 and 20% mmHg below the systolic BP^45^. Prior to the training session, a 5-min warm-up period involving stretching exercises was conducted by all participants. The selected exergame was boxing from XBOX360 Kinect Sports (Microsoft Game Studios, USA). Participants engaged in a 20-min competitive game play against each other. In the first week, seven participants were subjected to BFR using an Iranian-made inflatable cuff with a restriction level of 15 to 20% SBP (the cuff width employed was 6 cm), as determined by a sphygmomanometer. These participants played against seven competitors without BFR. After a seven-day interval, the training session was repeated, but the groups were switched in terms of BFR application. The cuffs were opened and closed at intervals of 5, 10, 15, and 20 min of games. The inflation and deflation cycles in the study consisted of 5-min intervals of boxing with BFR, followed by a one-minute rest period between game sets where BFR was not applied. Exercise intensity was monitored using the RPE scale. Specifically, participants indicated their RPE on the Borg 10-point scale at 5, 10, 15, and 20 min into the protocol^36^, providing numerical ratings to reflect their perceived effort.

Participants were instructed to provide self-reports of their perceived enjoyment levels in the activities using the Physical Activity Enjoyment Scale (PACES) developed by Kendzierski and DeCarlo^46^. The participants were asked to rate their enjoyment levels by indicating their typical feelings while engaging in physical activity. The scale employed a seven-point bipolar rating system, with response options ranging from “I enjoy it…I hate it”, “I feel bored…I feel interested”, to “It is enjoyable…It is very unpleasant”.

A Cardiac Holter monitoring system was used to measure HR. Prior to the commencement of the game, the Avicenna My Patch Holter device with four cables was affixed to the participants’ chests using f-55 SKINTACT chest leads manufactured in Austria and poly-gel ultrasound gel. This setup allowed for HR measurement during the game. To maintain a controlled testing environment, both the participants and assistants were instructed not to use mobile phones and were required to keep their devices at least 3 m away in the powered-off state. The leads were connected to the participants’ bodies following the standard procedures recommended by the manufacturer of the Holter system. Furthermore, the EE during exercise was calculated using the following formulas^47^:

$${\text{Male}}: \frac{(-55.0969+(0.6309 \times {\text{HR}})+(0.1988 \times \mathrm{ W}) +(0.2017 \times \mathrm{ A})) }{(4.184) \times 60 \times {\text{T}}},$$

$${\text{Female}}: \frac{(-20.4022 + (0.4472 \times \mathrm{ HR})- (0.1263 \times \mathrm{ W}) + (0.074\times {\text{A}})) }{(4.184) \times 60 \times \mathrm{ T}}.$$

HR—heart rate (beats per minute), W—weight (kg), A—age (years), T—time (length of exercise program in hours).

### Statistical analysis

Continuous variables are presented as mean ± standard deviation (SD). The normality of the data distribution of all variables was confirmed using the Shapiro–Wilk test and Q–Q plot. There were no missing values at any time point. A two-way analysis of variance (ANOVA) was conducted to evaluate the differences in the variables of EE, enjoyment, and interaction effects between exergame sessions with and without BFR. To examine the RPE data, the impact of the two exergame protocols, and sex on the RPE during the time, we employed Two-Way mixed ANOVA. All assumptions of two-way ANOVA and Two-way mixed ANOVA were checked. The analyses were conducted using SPSS (version 27, Armonk, NY, USA) and STATA version 17 (Stata Corp LLC, TX, USA), and p-values less than 0.05 were considered statistically significant.

## Results

**Figure 1:**
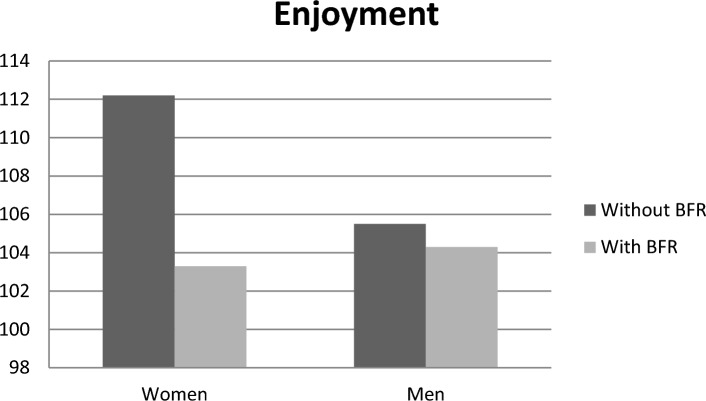
Comparison of enjoyment in two groups of men and women in two exergame protocols with and without BFR.

**Figure 2:**
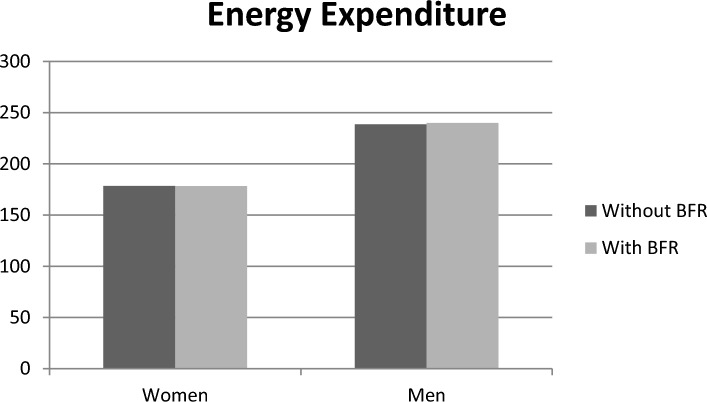
Comparison of energy expenditure in two groups of men and women in two exergame protocols with and without BFR.

The results of enjoyment revealed that the inclusion of BFR in the exergame led to a decrease in enjoyment for both men and women, compared to the exergame without BFR. However, this decrease was not statistically significant (F_1,25_ = 1.473, p = 0.236, ES = 0.056). Additionally, the comparison between women and men in both protocols did not show a significant difference (F_1,25_ = 0.390, p = 0.538, ES = 0.015), although women reported higher levels of enjoyment in the protocol with BFR (Fig. 1, Table 2). Regarding EE, the results indicated no significant difference between the two groups with and without BFR (F_1,25_ = 0.001, p = 0.977, ES = 0.001). However, a significant main effect for sex was observed for EE (F_1,25_ = 11.530, p = 0.002, ES = 0.316), with men in both protocols demonstrating significantly higher EE compared to women (Fig. 2). There was no interaction effect between sex and BFR for enjoyment (F_1,24_ = 0.682, p = 0.417, ES = 0.028) and EE (F_1,24_ = 0.001, p = 0.969, ES = 0.001).

**Table 2:** Descriptive statistics of parameters in protocols in men and women.

Variable	Without BFR		With BFR	
	Women	Men	Women	Men
Enjoyment	112.2 ± 9.46	105.5 ± 11.3	103.3 ± 12.2	104.3 ± 15.9
Energy expenditure	178.4 ± 27.9	238.6 ± 80.2	178.3 ± 36.4	239.9 ± 40.3
RPE				
0 min	1.30 ± 7.00	1.21 ± 6.67	2.35 ± 9.13	4.26 ± 10.8
5 min	2.64 ± 12.1	1.50 ± 11.3	1.12 ± 12.8	2.63 ± 15.8
10 min	2.32 ± 13.3	1.16 ± 12.8	2.33 ± 15	2.56 ± 15.8
15 min	1.48 ± 14.2	1.64 ± 13.5	1.51 ± 16	2.04 ± 16.8
20 min	2.56 ± 15.5	2.42 ± 14.6	1.68 ± 17.6	2.25 ± 17.6

Data are mean ± SD.

Table 2 presents the mean and standard deviation of the data for the two groups of women and men in the two protocols, with and without BFR. In terms of the reported RPE, women reported higher RPE values throughout the entire duration of the protocol without BFR, whereas, men reported higher RPE values at various time intervals in the protocol with BFR. However, none of these differences were significant (F_1,24_ = 3.37, p = 0.08, ES = 0.123).

Notably, both men and women reported higher values during all times in the protocol with BFR. However, this value was not significant (F_4,104_ = 0.19, p = 0.94, ES = 0.007). In addition, the comparison between women and men in both protocols (without and with BFR) did not yield a significant difference (F_4,104_ = 0.71, p = 0.59, ES = 0.026).

## Discussion

The results of the present study demonstrated that the inclusion of BFR in the exergame led to a decrease in reported enjoyment levels among both males and females, albeit without statistical significance. Additionally, no significant disparity in EE was observed between the two groups, one with BFR and the other without. However, a noteworthy finding emerged from the comparison of RPE, as both male and female groups reported significantly higher RPE values during the BFR session.

It has been established that enjoyment and RPE are inversely related, indicating that higher levels of enjoyment correspond to lower RPE scores^48^. RPE serves as a reliable indicator of exercise intensity and exhibits significant correlations between HR, respiration, and lactic acid accumulation, making it an essential measure in determining the intensity of physical activity^49^.

Previous studies have demonstrated that BFR exercises elicit heightened perceptual responses^26,30,38^. Furthermore, research has shown that aerobic exercise with BFR leads to higher levels of perceived exertion, oxygen consumption, total blood lactate, and HR when compared to aerobic exercise performed at the same relative intensity without BFR^33^. These effects are likely attributed to the elevated metabolic stress induced by BFR, suggesting that low-intensity aerobic exercise with BFR may yield mood state responses similar to those observed during high-intensity aerobic exercise without BFR^33^. A study conducted by Suga et al. supports our findings, as they reported lower levels of enjoyment and higher perceived pressure following BFR training compared to training without BFR^50^. Additionally, other studies have also reported similar results regarding perceived pressure, corroborating the findings of our study^27,34^. The investigation conducted by da Silva et al. showed that both aerobic exercise with BFR and high-intensity interval exercise without BFR resulted in comparable increases in fatigue and tension while inducing greater decrements in mood states compared to the control aerobic exercise condition^33^. These findings highlight the influence of BFR on this particular physiological response^33^. In a separate study conducted by Mok et al., it was observed that RPE and leg discomfort were significantly higher during BFR walking compared to walking without BFR. However, immediate enjoyment following the walking activity was significantly lower when BFR was utilized^38^. Additionally, Silva et al. found that mood states were reduced after low-intensity resistance training with BFR, whereas no such reduction was observed after low-intensity resistance training without BFR^35^. Moreover, other investigations have reported that perceptual responses induced by low-intensity aerobic exercise with BFR were greater than those induced by low-intensity aerobic exercise without BFR and similar to those induced by high-intensity aerobic exercise^32,33^. Consequently, high-intensity exercise leads to heightened perceptual responses, including increased perceived exertion and decreased effect, which may be perceived as barriers to engaging in physical activity. It is plausible that affective responses during exercise are modulated by HR and perceived exertion. Thus, the outcomes related to cardiovascular responses, especially HR, during BFR walking may contribute to elucidating its adverse impact on perceived exertion and affective responses. Alterations in exercise-induced perceptual parameters may be associated with metabolic and cardiovascular responses, such as increased metabolite levels in the blood and skeletal muscle^38^.

BFR training has emerged as a technique that influences muscle metabolism and fatigue when compared to performing the same exercise without occlusion^34,51^. The application of pressure using a restrictive cuff reduces arterial inflow and impedes venous clearance from the exercising limb. In terms of neuromuscular drive, this restriction in oxygen supply and loss of contraction efficiency may result in increased overall muscle activity, thereby stimulating the recruitment of high-threshold motor units. Consequently, this heightened muscle activity leads to an increased anaerobic metabolic demand and subsequent production of metabolites. The hampered venous metabolic clearance ultimately causes the accumulation of metabolic byproducts in the limb distal to the cuff^52^. Hollander et al. suggest that ischemic pain, coupled with decreased metabolite clearance and artery deformation, could intensify the perception of pain and exertion, respectively^53^. For instance, Suga et al.^34^ observed a significant depletion of creatine phosphate, as well as decreases in dihydrogen phosphate and pH levels resulting from both low- and high-intensity resistance exercises with and without BFR, respectively. Notably, changes in pH are particularly significant as a reduction in intramuscular pH is known to activate afferent nerve fibers via muscle chemoreceptors, thus elevating pain sensitivity and potentially negatively affecting mood states^54–57^. Additionally, metabolite accumulation may contribute to peripheral fatigue by impairing calcium kinetics and, consequently, restraining actin–myosin interaction^58^. This likely contributes to the increased peripheral fatigue reported in the current investigation^33^. It remains unclear whether the perceived discomfort associated with wearing BFR-restricting cuffs during exercise influenced participants’ perceptual responses. However, in our study, it is important to note that the non-significant decrease observed in reported enjoyment values may indicate the positive impact of excitement and competition induced by playing exergames. This, in turn, mitigates the negative effects of cuff closure and BFR, thereby increasing the likelihood of participating in these activities. Moreover, the combination of exergames with BFR demonstrates a more favorable effect.

The comparison of reported enjoyment between women and men did not reveal a statistically significant difference in both protocols; however, women reported higher enjoyment levels in both conditions. Previous research has highlighted gender disparities in exercise-related motivation and enjoyment^59,60^. Numerous studies have demonstrated the importance of gender in the context of physical activity enjoyment^61,62^. It is worth noting that inconsistent outcomes in the literature investigating enjoyment of exergaming by sex may be influenced by the specific exergames employed. For instance, Ferreira et al. reported sex differences in enjoyment, with females experiencing greater enjoyment than males during active video game activities, although no differences were observed in EE^39^. Similarly, a study conducted by McDonough et al. suggested that exergaming sessions elicited significantly higher enjoyment and RPE compared to treadmill sessions, particularly among women^15^. In terms of EE, a significant difference was observed between the two groups of women and men, with men displaying significantly higher EE in both protocols^15^. Notably, significant gender differences in EE during exergames have been observed in studies involving children, adolescents, and adults^63–67^. A previous study showed significantly higher EE in older men compared to older women^64^. Some studies have reported that girls exhibit lower levels of physical activity than boys during exergaming play^68,69^, while others have found no differences^40,70,71^.

In the present study, the comparison of RPE shown in men and women did not yield a significant difference in both protocols. However, within the protocol involving BFR, men reported higher RPE values at various time intervals compared to women. As BFR induces muscle pain and discomfort during exercise, which can contribute to elevated RPE^72^, and considering the substantial increase in HR observed in men during BFR exergaming, this finding suggests a higher training intensity in the BFR protocol for men. Previous research has shown that men exhibit a significant increase in epinephrine levels in response to acute psychological stress, while women either experience no increase or a slight increase^73^. A similar but smaller gender difference has also been observed for norepinephrine, with men displaying more pronounced changes in hormone levels. These differences imply that men’s and women’s responses to mental stress may involve distinct mechanisms. Gender differences in cardiovascular responses and adaptations to exercise could potentially influence vascular function in response to exercise with BFR^74^. Some studies have suggested that pressure thresholds and pain tolerance may differ between sexes in the context of ischemic exercise^75,76^. However, Spitz et al. have found no gender disparities in ratings of perceived pain in response to ischemia^77^. Nevertheless, when employing alternative pain measurement methods such as pain pressure threshold and pain tolerance, these same researchers discovered that men exhibit greater pain tolerance and pressure threshold than women. Hence, variations in types of perceived discomfort may contribute to discrepancies in findings across studies^77^.

## Limitations and future directions

Several limitations should be acknowledged in this research. Firstly, the relatively small sample size may constrain the generalizability of the findings to a broader population, as the diminutive sample may not adequately represent the larger community, thereby limiting the broader applicability of the study’s outcomes. Another limitation is the lack of control over the psychological conditions and motivation of the participants. Furthermore, the influence of the high excitement of the environment, the specific game type, and the competitive nature of the two-person setting should also be considered as potential limitations. The scope of applicability to diverse demographic profiles is hindered by the homogeneity of the participant sample, comprising exclusively young individuals with similar age distributions and BMIs. It is important to emphasize that the findings of this study only reflect short-term responses immediately after exercise. Therefore, the exploration of long-term effects should be a priority for future investigations. While existing evidence supports the effectiveness of BFR in enhancing training intensity, the role of gender in the observed responses and subsequent exercise adaptations has received less attention. Finally, due to the great popularity of these video games in different generations, to increase mobility, more studies to improve the exercise aspects of these types of entertainment and their effects on various physical and psychological factors should be given more attention by researchers. Future studies utilizing more diverse cohorts should aim to investigate the long-term effects of combining BFR with exergames to gain a comprehensive understanding of its impact.

## Practical applications

The addition of BFR to exergame decreased the enjoyment values reported in both male and female groups. Also, more perceived pressure was reported by both men and women. However, the amount of EE did not show a significant difference compared to the without BFR. Of course, the gender comparison showed that the EE of men was more than women. The importance of these data is that although adding BFR can intensify some physiological responses, it can still affect people’s enjoyment and reduce adherence to it in the long term. Examining the effect of gender can also reveal in which sectors differences exist and in which circumstances they can be ignored. This contributes to the provision of specialized programs for women and males. If we can apply new methods such as BFR to low-moderate activities while maintaining their enjoyment for long-term adherence, we can increase the efficacy of these activities on both acute responses and long-term adaptations to exercise.

## Conclusion

Exergames incorporating BFR have an impact on perceptual responses, specifically enjoyment, and perceived pressure. Additionally, gender comparison revealed a significant difference in EE, with men displaying higher values.

## Data Availability

The datasets used and/or analyzed during the current study are available from the corresponding author upon reasonable request.

## Abbreviations

ANOVA: Analysis of variance
BFR: Blood flow restriction
BP: Blood pressure
EE: Energy expenditure
HR: Heart rate
PACES: Physical activity enjoyment scale
RPE: The rating of perceived exertion
